# Prevention and management of ventilator-associated pneumonia: A survey on current practices by intensivists practicing in the Indian subcontinent

**DOI:** 10.4103/0019-5049.79889

**Published:** 2011

**Authors:** Deven Juneja, Omender Singh, Yash Javeri, Vikas Arora, Rohit Dang, Anjali Kaushal

**Affiliations:** Department of Critical Care Medicine, Max Super Speciality Hospital, Saket, New Delhi, India

**Keywords:** Intensivists, knowledge, attitude and practices study, VAP bundles, ventilator-associated pneumonia

## Abstract

Implementation of evidence-based guidelines to prevent and manage ventilator-associated pneumonia (VAP) in the clinical setting may not be adequate. We aimed to assess the implementation of selected VAP prevention strategies, and to learn how VAP is managed by the intensivists practicing in the Indian Subcontinent. Three hundred 10-point questionnaires were distributed during an International Critical Care Conferenceheld at New Delhi in 2009. A total of 126 (42%) questionnaires distributed among delegates from India, Nepal and Sri Lanka were analyzed. Majority (96.8%) reported using VAP bundles with a high proportion including head elevation (98.4%), chlorhexidine mouthcare (83.3%), stress ulcer prophylaxis (96.8%), heat and moisture exchangers (HME, 92.9%), early weaning (94.4%), and hand washing (97.6%) as part of their VAP bundle. Use of subglottic secretion drainage (SSD, 45.2%) and closed suction systems (CSS, 74.6%) was also reported by many intensivists, whereas use of selective gut decontamination was reported by only 22.2%. Commonest method for sampling was endotracheal suction by 68.3%. Gram negative organisms were reported to be the most commonly isolated. Majority (39.7%) reported using proton pump inhibitors for stress ulcer prophylaxis and 84.1% believed that VAP contributed to increased mortality. De-escalating therapy was considered in patients responding to treatment by 57.9% and 65.9% considered adding empirical methicillin resistant *Staphylococcus* aureus (MRSA)coverage, while 63.5% considered adding nebulized antibiotics in certain high-risk patients. There was good concordance regarding VAP prophylaxis among the intensivists with a majority adhering to evidence-based guidelines. We could identify certain issues like the choice of agent for stress ulcer prophylaxis, use of HME filters, SSD and CSS, where there still exists some practice variability and opportunities for improvement.

## INTRODUCTION

Ventilator-associated pneumonia (VAP) is the most common nosocomial infection among the critically ill patients admitted in the intensive care units (ICUs),[1,2] and is associated with increased mortality rate, hospital length of stay and costs for patients who acquire it.[3] Prevention of VAP is given paramount importance in all quality control programs as it may help in improving clinical outcome and reduce costs. Moreover, there is an increased rate of VAP caused by multi-drug resistant strains in the recent years which may further add on to mortality and morbidity.[4] In spite of this, implementation of the available international evidence-based guidelines and recommendations to prevent and manage VAP into the clinical setting may not be adequate leading to sub-optimal patient care and increased VAP rates.[5] Hence, we conducted this survey to assess the implementation of selected VAP prevention strategies, and to learn how VAP is managed by the intensivists practicing in the Indian subcontinent.

## METHODS

The issues pertaining to the prevention and management of VAP were identified and formulated in a 10-point questionnaire form. Questions covering the following aspects regarding the prevention and management of VAP were prepared:

- Use and components of VAP bundles
- Diagnosis of VAP
- Treatment of VAP

1. Do you employ VAP bundle in your ICU?
  - Yes
  - No
2. Which of the following are the components of your VAP bundle
  - 30–45% head elevation
  - Chlorhexidine mouthcare
  - Selective gut decontamination
  - Stress ulcer prophylaxis
  - Daily wake tests
  - Use of subglottic secretion drainage (SSD) endotracheal tube
  - Closed suction systems (CSS)
  - Heat and moisture exchangers (HMEs)
  - Early weaning
  - Hand washing
3. Method for sampling used for diagnosis of VAP
  - Endotracheal suction
  - Mini broncho-alveolar lavage (BAL)
  - BAL
  - Protectedspecimen brush (PSB)
4. Which is the most commonly isolated organism in your ICU
  - Staphylococcus aureus
  - Pseudomonas aeruginosa
  - *Klebsiella* spp
  - Acinetobacter baumannii
  - Escherichia coli
  - *Candida* spp
  - Others, please specify
5. Which agent do you use for stress ulcer prophylaxis
  - Sucralfate
  - H_2_ receptor blockers
  - Proton pump inhibitors
  - Combination
6. Do you feel VAP contributes to increased mortality in your ICU?
  - Yes
  - No
7. Duration of therapy you employ for treatment of VAP
  - Up to 7 days
  - 7 - 14 days
  - More than 14 days
  - Till clinical improvement
8. In which patients do you consider de-escalation of therapy?
  - All patients
  - Only those with clinical improvement
  - None
9. When do you consider adding empirical coverage for methicillin resistant *Staphylococcus aureus* (MRSA)
  - In all patients with suspected VAP
  - Certain high-risk patients
  - Never
10. When do you consider adding nebulized antibiotics?
  - In all patients with suspected VAP
  - Certain high-risk patients
  - Never

Three hundred questionnaires were distributed to the delegates during an International Conference of Critical Care Medicine conducted by Asia Pacific Critical Care society in December 2009 (APCC 2009) in New Delhi. The answered questionnaires were collected back at the end of the conference.

Intensivists were defined as general physicians, pulmonologists or anaesthesiologists who were currently involved in taking care of critically ill patients in ICUs. Postgraduate students in training in the field of intensive care also answered some of the questionnaires.

## RESULTS

Out of the 300 questionnaires distributed, 131 (43.7%) were returned. Out of these 131, five were excluded from the final analysis as two each belonged to delegates from Saudi Arabia and Hong Kong and one was from a delegate from London. One hundred and twenty-six (96.2%) responses from the delegates of India, Nepal and Sri Lanka, were included in the final analysis. Maximum number of respondents were practicing in New Delhi (42.9%), followed by Haryana (10.3%) [Table 1]. On categorizing the surveyed intensivists according to their base specialty, 67 (53.2%) were from anaesthesia, 36 (28.6%) were from general medicine and 23 (18.3%) from pulmonology.

**Table 1:** Distribution of respondents according to their region of practice

Andhra Pradesh	**4 (3.2)**
Delhi	**54 (42.9)**
Haryana	**13 (10.3)**
Karnataka	**5 (4)**
Kerala	**1 (0.8)**
Maharashtra	**12 (9.5)**
Orissa	**3 (2.4)**
Punjab	**5 (4)**
Rajasthan	**2 (1.6)**
Uttar Pradesh	**12 (9.5)**
Uttarkhand	**2 (1.6)**
West Bengal	**4 (3.2)**
Srilanka	**5 (4)**
Nepal	**4 (3.2)**

Figures in parentheses are in percentage

Most of the intensivists (96.8%), reported using VAP bundles in their ICUs with a high proportion reporting including head elevation (98.4%), chlorhexidine mouthcare (83.3%), stress ulcer prophylaxis (96.8%), heat and moisture exchangers (92.9%), early weaning (94.4%) and hand washing (97.6%) as part of their VAP bundle. Use of SSD (45.2%) and CSS (74.6%) was also reported by many intensivists, whereas use of selective decontamination of the digestive tract (SDD) was reported by only 22.2% of respondents. Most common method for sampling used for diagnosis of VAP was endotracheal suction by 86 (68.3%) intensivists, and only 0.8% intensivists reported using protected-sample brush. Gram negative organisms (*Pseudomonas, Acinetobacter*) were reported to be the most commonly isolated organisms. Majority of respondents (39.7%) reported using proton pump inhibitors for stress ulcer prophylaxis. Most of the intensivists (84.1%) believed that VAP contributed to increased mortality in their ICUs with 47.6% treating VAP with an antibiotic course lasting for 7–14 days. De-escalating therapy was considered in patients responding to treatment, by 57.9% and 65.9% considered adding empirical MRSA coverage, while 63.5% considered adding nebulized antibiotics in certain high-risk patients [Table 2].

**Table 2:** Response to various questions regarding management of VAP

Question	Response	Number (%)
Do you employ VAP bundle in your ICU?	Yes	122 (96.8)
	No	4 (3.2)
Which of the following are the components of your VAP bundle	30 – 45% head elevation	124 (98.4)
	Chlorhexidine mouthcare	105 (83.3)
	Selective gut decontamination	28 (22.2)
	Stress ulcer prophylaxis	122 (96.8)
	Daily wake tests	92 (73)
	Use of subglottic drainage	57 (45.2)
	Closed suction devices	94 (74.6)
	HME filters	117 (92.9)
	Early weaning	119 (94.4)
	Hand washing	126 (97.6)
Method for sampling used for diagnosis of VAP	ET suction	86 (68.3)
	Mini BAL	11 (8.7)
	BAL	28 (22.2)
	PSB	1 (0.8)
Which is the most commonly isolated organism in your ICU*	*Staphylococcus aureus*	20 (15.9)
	*Pseudomonas*	54 (42.9)
	*Klebsiella*	25 (19.8)
	Acinetobacter	52 (41.3)
	*E. coli*	13 (10.3)
	*Candida*	8 (6.3)
Which agent do you use for stress ulcer prophylaxis	Sucralfate	8 (6.3)
	H_2_ receptor blockers	22 (17.5)
	Proton pump inhibitors	50 (39.7)
	Combination	45 (35.7)
Do you feel VAP contributes to increased mortality in your ICU?	Yes	106 (84.1)
	No	20 (15.9)
Duration of therapy you employ for treatment of VAP	Upto 7 days	7 (5.6)
	7 – 14 days	60 (47.6)
	More than 14 days	6 (4.8)
	Till clinical improvement	53 (42.1)
In which patients do you consider de-escalation of therapy?	All patients	53 (42.1)
	Only those with clinical improvement	73 (57.9)
	None	0
When do you consider adding empirical coverage for MRSA	In all patients	28 (22.2)
	Certain high-risk patients	83 (65.9)
	Never	14 (11.1)
When do you consider adding nebulized antibiotics?	In all patients	2 (1.6)
	Certain high-risk patients	80 (63.5)
	Never	43 (34.1)

* A few intensivists believed that more than one organisms were commonly isolated from their lower respiratory tract samples, and hence, they were allowed to mark more than one option, VAP - Ventilator-associated pneumonia, ICU - Intensive care unit, MRSA - Methicillin resistant *Staphylococcus aureus*

A few intensivists did not answer some questions but their number was not significant enough to change the results. A few intensivists believed that more than one organism were commonly isolated from their lower respiratory tract samples, and hence, they were allowed to mark more than one option.

## DISCUSSION

We conducted this survey with the aim of evaluating the current practices regarding prophylaxis and management of VAP among the intensivists in the Indian subcontinent. Such surveys may be useful in identifying the shortcomings and detect areas which require improvement. Through this study we could demonstrate that even though most of the intensivists adhered to the recommended practices regarding most of the issues pertaining to management of VAP, there still existed some ambiguity on certain other issues like the choice of agent for stress ulcer prophylaxis, use of HME, SSD and closed suction systems.

The prevention of VAP is a vital component in the management of critically ill patients requiring mechanical ventilation. To achieve this aim many measures have been evaluated and recommended.[4,6] Incorporation of a set of evidence-based practices to prevent VAP, called the VAP bundles, may reduce the incidence of VAP in mechanically ventilated patients. In addition, incorporation of these VAP bundles in the clinical practice may result in decreased ventilator days, ICU stay, and mortality rates.[7] The importance of VAP bundles was recognized by a high proportion (96.8%) of intensivists in our cohort who had incorporated it in their everyday practice.

VAP may develop from aspiration of oral secretions from area around the tube cuff. Hence, measures likely to reduce such aspiration like limiting the use of sedative and paralytic agents that depress cough and other host-protective mechanisms, maintaining adequate endotracheal cuff pressure (more than 20 cm H_2_O), and use of continuous aspiration of subglottic secretions, through specially designed endotracheal tubes, may all significantly reduce the incidence of VAP[8–10] and are recommended by International guidelines.[4,6,11,12] SSD is a simple measure that reduces chronic micro-aspirations from area around the cuff of endotracheal tubes and hence aid in VAP prevention. A large meta-analysis revealed that application of SSD reduces the incidence of VAP by nearly half, primarily by reducing early-onset pneumonia, reduces the duration of mechanical ventilation by 2 days and the length of ICU stay by almost 3 days. In patients who developed VAP, use of SSD delayed the onset of VAP by 6.8 days.[10] In addition, it has been recommended to keep the patient head elevated at 30–45° as supine positioning may also facilitate aspiration.[13,14] Apart from the use of SSD, which was incorporated in VAP bundles by only 45.2%, all the other measures including head elevation (98.4%), daily wake tests (73%) and early weaning from mechanical ventilation (94.4%) found wide acceptance among the surveyed intensivists.

Use of CSS to prevent VAP is a contentious issue. Although it may have some theoretical advantage over open suction system with less chance of external contamination, several meta-analytical reviews have failed to show any benefit in terms of reduced VAP rates, ICU length of stay or mortality.[15–17] Hence, current evidence does not support use of CSS to prevent VAP and reasons other than VAP prevention should determine the choice of the suction system.[4,6,15] In spite of these recommendations, 74.6% intensivists in our cohort reported using CSS to prevent VAP.

A great majority of intensivists (92.9%) in our survey reported using HME for VAP prevention. Passive humidifiers or heat–moisture exchangers reduce colonization of the ventilator circuit and hence may have a role in VAP prevention. The American Thoracic Society[4] and Center for Disease Control (CDC) guidelines[6] do not recommend use of HME filters for VAP prevention as their use has not consistently shown to reduce the incidence of VAP.[18,19]

A recent meta-analysis showed that oral decontamination of mechanically ventilated patients using antiseptics was associated with a lower risk of VAP but it did not have any effect on duration of mechanical ventilation, ICU stay or overall mortality.[20] Another meta-analysis reported that use of topical chlorhexidine resulted in a reduced incidence of VAP (relative risk, 0.74) with maximum benefit in cardiac surgery patients. This meta-analysis too failed to show any mortality benefit with chlorhexidine oral care.[21] Based on the emerging evidence, recent guidelines recommend use of oral antiseptic rinses for VAP prevention.[11,12] A great majority of respondents (83.3%) reported using chlorhexidine mouth care in their ICU in keeping with the current guidelines.

Even though SDD may lead to lower incidence of VAP with higher ICU survival, and has helped contain outbreaks of multi-drug resistant bacteria,[22,23] it may increase antibiotic resistance in microorganisms[24,25] and it should be used selectively to control outbreaks and is currently not recommended for routine use.[4,11] In accordance with these recommendations, only 22.2% intensivists have incorporated SDD in their daily routine practice as a part their VAP bundle.

As healthcare personnel may aid in spread of infection from patient to patient by inadequate hand hygiene, it is strongly recommended that all ICU staff must decontaminate hands by washing them with either soap and water or by using an alcohol-based antiseptic agent.[6] Proper hand hygiene was perceived as an important measure to prevent VAP and was incorporated in their respective VAP bundles by 97.6% of the intensivists who returned our questionnaire.

Patients on mechanical ventilators are routinely given stress ulcer prophylaxis as these patients are at increased risk for gastrointestinal bleeding. Most commonly employed agents include H2 receptor blockers, sucralfate and proton pump inhibitors. In a comparative study, in critically ill patients on mechanical ventilation, there were no significant differences in VAP rates, duration of ICU stay, or mortality among those receiving ranitidine or sucralfate.[26] Nevertheless, patients receiving ranitidine had a significantly lower rate of gastrointestinal bleeding than those treated with sucralfate.[26] The guidelines suggest that any of these agents can be used for stress ulcer prophylaxis.[4,6] In our study, the most commonly used agents were proton pump inhibitors reported to be used by 39.7% intensivists. Presently, there is inadequate data regarding the role of proton pump inhibitors in VAP prevention. On the contrary, evidence suggests that these agents may increase the risk of *Clostridium difficile* disease, and hence, they are not currently recommended.[4,6,12]

Guidelines recommend that in all patients with suspected VAP quantitative cultures of lower respiratory secretions should be taken before administration of antibiotic therapy, to define both the presence of pneumonia and the etiologic pathogen.[4] If bronchoscopic sampling is not immediately available, therapy should not be postponed for the purpose of performing diagnostic studies as delay in the initiation of appropriate antibiotic therapy can lead to increased mortality.[4] Alternatively, non-bronchoscopic methods like endotracheal aspiration or mini-BAL can be performed to reliably obtain lower respiratory tract secretions.[4] Our study demonstrated that most of the intensivists (68.3%) preferred endotracheal aspiration as the method to obtain lower respiratory secretions as it is easy to perform, minimally invasive, does not require any expertise and is widely available. Not surprisingly, less than 1% intensivists preferred to perform PSB routinely to diagnose VAP.

There are significant differences in the pathogens responsible for infection and their susceptibility patterns from one hospital to another.[27] In general, the most frequently involved pathogens include Gram-negative bacteria like *Pseudomonas aeruginosa, Acinetobacter baumannii*, and *Klebsiella spp*.[4] Emergent evidence suggests increased incidence of infection due to gram-positive cocci, such as *Staphylococcus aureus*, particularly methicillin resistant strains in *Staphylococcus aureus*(MRSA).[28] Patients with diabetes mellitus, head trauma, altered sensorium and those admitted in ICUs are especially more prone to develop pneumonia due to *S. aureus*.[29] The respondents in our study also identified gram-negative organisms like *P. aeruginosa, Acinetobacter* speciesand *K. pneumonia* as the most frequently isolated organisms from patients with VAP, which is in accordance with the world literature.

As there is wide variability in the pathogens responsible and their sensitivity patterns, the choice of antimicrobial drug should vary depending upon the patient factors, suspected pathogen and local epidemiology.[27] Moreover, as the patients with prolonged intubation periods and prior use of antibiotics are at high risk of developing MRSA pneumonias[29] it may be advisable to add empirical MRSA coverage in this sub-group of patients. Nevertheless, early de-escalation should be considered depending on the subsequent microbiological results.

A 10-point, evidence-based practice guideline for the treatment of VAP, the Tarragona strategy highlights the four basic principles of VAP management including initial broad spectrum antibiotic coverage with de-escalation when indicated, high and individualized doses of antibiotics based on local and patient factors, early administration of antimicrobial treatment, and choice of antimicrobial agent with regard to lung penetration.[30] Patient’s clinical response should dictate the duration of therapy and antibiotic therapy should be de-escalated and ultimately stopped as soon as possible to minimize the risk of bacterial resistance.[31] In the present survey, 42.1% intensivists agreed that the duration of their antibiotic therapy was guided by the patient’s clinical response and most of them (47.6%) employed an antibiotic course of 7-14 days duration. Only 4.8% intensivists considered it judicious to give a prolonged antibiotic course of more than 14 days. A good proportion (65.9%) of intensivists surveyed also agreed to adding empirical MRSA coverage in certain high-risk patients developing VAP.

There are certain inherent advantages of using nebulized antibiotics including attaining high antibiotic concentrations at the infection site with low systemic absorption, thereby avoiding systemic toxicity which may be important particularly for certain nephrotoxic drugs like aminoglycosides or vancomycin. Aminoglycosides, polymyxins and vancomycin have also been used in nebulized forms.[32–34] A recently published study showed that, nebulized gentamicin combined with intravenous antibiotics led to a lower VAP rate, less bacterial resistance and use of fewer systemic antibiotics and might have a role in early weaning from mechanical ventilation.[33] Nebulized colistin has also been tried in multi-drug resistant Gram-negative pneumonia with good results.[34] The main drawback for using this technique is inadequate drug dispersal in mechanically ventilated patients leading to deposition of drug in the ventilator circuits and the tracheobronchial tree. Hence, routine use of aerosolized antibiotics to treat VAP is not recommended but it can be used as an add-on treatment of multidrug-resistant gram-negative infections.[35]

### Limitations

It is obvious that a short 10-point questionnaire cannot cover all issues pertaining to VAP prevention and management. In addition, as the conference, in which the survey was conducted, was held in New Delhi, India, most of the respondents were also from Delhi and other nearby cities. Hence, other cities and especially other countries like Nepal and Sri Lanka might not be adequately represented.

## CONCLUSIONS

There was good concordance regarding VAP prophylaxis among the intensivists with a majority adhering to evidence-based recommendations and guidelines. Even though the gap between recommended guidelines and the actual clinical practice is closing, we could identify certain popular measures like the choice of agent for stress ulcer prophylaxis, use of HME and closed suction systems which require further validation regarding their role in VAP prevention through larger trials. In addition, use of SSD, which is still not widely accepted and utilized, should be promoted through educational programs, formulation of local protocols and ensuring availability of these specialized endotracheal tubes.
